# Cabozantinib, a Multityrosine Kinase Inhibitor of MET and VEGF Receptors Which Suppresses Mouse Laser-Induced Choroidal Neovascularization

**DOI:** 10.1155/2020/5905269

**Published:** 2020-06-19

**Authors:** Xiaoli Zhang, Manhui Zhu, Laiqing Xie, Xiaodong Sun, Jiaowen Xu, Yang Guo, Dong Liu, Yunwei Shi, Xun Xu, E Song

**Affiliations:** ^1^Department of Ophthalmology, The Second Affiliated Hospital of Soochow University, Suzhou, Jiangsu, China; ^2^Department of Ophthalmology, Lixiang Eye Hospital of Soochow University, Suzhou, Jiangsu, China; ^3^Shanghai Key Laboratory of Ocular Fundus Disease, Shanghai, China; ^4^Department of Ophthalmology, Shanghai First People's Hospital, School of Medicine, Shanghai JiaoTong University, Shanghai, China; ^5^Coinnovation Center of Neuroregeneration, Jiangsu Key Laboratory of Neuroregeneration, Nantong University, Nantong, China

## Abstract

Choroidal neovascularization (CNV) is a leading cause of blindness in the elderly in developed countries and is particularly associated with age-related macular degeneration (AMD). Cabozantinib (CBZ) hinders the activation of multiple receptor tyrosine kinases involved in tumor angiogenesis, such as hepatocyte growth factor receptor (MET) and vascular endothelial growth factor receptor 2 (VEGFR2). We aimed to investigate the role and mechanism of CBZ in a mouse laser-induced CNV model. In zebrafish embryos, CBZ perturbed intersegmental vessel (ISV) formation without obvious neurodevelopment impairment. In the mouse laser-induced CNV model, phosphorylated hepatocyte growth factor receptor (p-MET) and phosphorylated vascular endothelial growth factor receptor 2 (p-VEGFR2) were increased in the CNV region. CBZ intravitreal injection or oral gavage alleviated CNV leakage and the CNV lesion area without obvious intraocular toxicity, as well as disturbed the phosphorylation of MET and VEGFR2. Additionally, CBZ downregulated the expression of the hepatocyte growth factor (HGF) with no effect on the expression of the vascular endothelial growth factor (VEGF). CBZ downregulated HGF, p-MET, and p-VEGFR2 expressions *in vitro*, as well as inhibited the proliferation, migration, and tube formation of b-End3 cells. In summary, CBZ alleviates mouse CNV formation possibly via inhibiting the activation of MET and VEGFR2. The findings provide a novel potential therapy method for CNV patients.

## 1. Introduction

Choroidal neovascularization (CNV) is a leading cause of blindness in the elderly in the developed world, especially because CNV is related to age-related macular degeneration (AMD) [1]. CNV is characterized by the formation of new pathogenic vessels in the choroid that breach Bruch's membrane (BM) and enter the subretinal space [2]. Current treatment strategies for CNV have their shortcomings. For example, although laser photocoagulation preserves central vision, it also leads to peripheral visual loss [3]. Moreover, antivascular endothelial growth factor (anti-VEGF) drugs to treat CNV are only effective in some, and safety concerns have emerged regarding long-term side effects such as the degeneration of normal blood vessels, the neural retina, and the choroid [4]. Thus, safer therapeutic agents are needed for the effective treatment of vision-threatening CNV.

Cabozantinib (CBZ) is a tyrosine kinase inhibitor (TKI) that downregulates the activation of multiple receptor tyrosine kinases involved in tumor angiogenesis, invasion, and metastasis, including hepatocyte growth factor receptor (MET) and vascular endothelial growth factor receptor 2 (VEGFR2) [5, 6]. The MET ligand hepatocyte growth factor (HGF) plays a vital role in promoting breast cancer progression and metastasis [7]. Upon binding to MET, HGF induces multiple biological responses inside cancer cells, leading to enhanced cell migration, matrix degradation, invasiveness, and angiogenesis [8, 9]. Tyr1234 and Tyr1235 are critical for the activation of MET in the reaction with the ligand in intact cells [10]. Although the pathogenesis of CNV is extremely complex, numerous basic and clinical studies have revealed that VEGF is a key mediator in the pathogenesis of CNV [11]. VEGF regulates endothelial cell behavior by binding to three types of VEGF receptors (VEGFRs), VEGFR1, VEGFR2, and VEGFR3. Among these receptors, VEGFR2, which is mainly expressed on endothelial cells, is responsible for mediating the angiogenic effects of VEGF [12]. Upon binding to VEGF, VEGFR2 autophosphorylates its cytoplasmic tyrosine residues, which promotes tyrosine phosphorylation of several signal transduction proteins, thereby initiating the downstream signaling pathways of VEGF-induced endothelial cell proliferation, migration, and morphogenesis [13]. Furthermore, the phosphorylation of Y951 inside VEGFR2 is associated with tumor angiogenesis [14, 15].

In the present study, we explored whether CBZ alleviated CNV formation via inhibiting the activation of MET and VEGFR2. In zebrafish embryos, CBZ perturbed intersegmental vessel (ISV) formation without neurodevelopment impairment. In a mouse laser-induced CNV model, p-MET and p-VEGFR2 were increased in the CNV region. CBZ intravitreal injection or oral gavage alleviated CNV leakage and the CNV lesion area without intraocular toxicity and disturbed the phosphorylation of MET and VEGFR2. CBZ downregulates p-MET and p-VEGFR2 expressions *in vitro*, as well as inhibits proliferation, migration, and the tube formation of b-End3 cells. The findings of this study provide a novel potential therapy method for patients with CNV.

## 2. Materials and Methods

### 2.1. Zebrafish and Drug Treatment

The study was performed according to the local institutional laws and the Chinese law for the Protection of Animals. We followed the methods of Li et al. [16]. The following transgenic zebrafish types were used: Tg (Flk:mcherry: Hb9:EGFP), which expresses mCherry (red fluorescence) under the control of Flk (also termed vascular endothelial growth factor receptor 2, a vascular endothelial cell marker) and EGFP (green fluorescence) under the control of Hb9 (also termed motor neuron and pancreas homeobox 1, a differentiated motor neuron marker), and Tg (Flk:EGFP), which expresses EGFP (green fluorescence) under the control of the regulatory sequences of Flk. At 6 hpf (hours post fertilization), the embryos were scanned by anatomical microscopy to exclude morphologically aberrant individuals. Approximately 10 healthy embryos/well were placed in a 24-well plate with E3 solution. At 8 hpf, the E3 solution was replaced with CBZ (Cometriq, Exselixis Inc., San Francisco, CA, USA; dissolved in 0.1% DMSO) treatment solution at different concentrations (0.01, 0.1, 1, 5, 10, 50, 100, and 500 *μ*g/ml). At 48 hpf, the zebrafish embryos were gathered and fixed with 4% paraformaldehyde (PFA) diluted in phosphate-buffered saline (PBS) for intersegmental vessel (ISV) and neuron imaging.

### 2.2. Imaging

At 48 hpf, the zebrafish embryos were anesthetized with E3 solution/0.16 mg/ml tricaine (Sigma-Aldrich, St. Louis, MO, USA) and were embedded in 0.8% low melt agarose. Confocal imaging was performed using a confocal microscope (Zeiss 510 META, Carl Zeiss Micro-Imaging, USA).

### 2.3. Mouse Laser-Induced CNV Model and Drug Treatment

Six-to-eight-week-old male C57B/L6 mice (Experimental Animal Center, Soochow University, Suzhou, Jiangsu, China) were used to generate the laser-induced CNV model [17]. Three days following CNV, anesthetized mice received a single intravitreal injection of 1 *μ*l of CBZ solution (2 *μ*g/*μ*l in 0.1% DMSO), 1 *μ*l of ranibizumab, a recombinant humanized monoclonal antibody fragment binding all active isoforms of VEGF-A (RBZ; Lucentis; Novartis, Basel, Switzerland), or an equivalent volume of 0.1% DMSO. Four days later, the mice were sacrificed for further experiments. For the oral gavage experiment, CBZ was suspended in a solution containing 0.5% carboxymethylcellulose (CMC) (Sigma-Aldrich) and 5% glucose, and the mice received by oral gavage (once daily) the CBZ suspension (200 or 300 mg/kg per day) or an equivalent volume of solution containing 0.5% CMC and 5% glucose. Oral gavage began on the day of CNV performance and lasted for 2 weeks before analysis.

### 2.4. Western Blotting

The ocular tissues including the retinal pigment epithelium (RPE) and choroid mix were isolated from enucleated eyes using a dissecting microscope to perform western blotting [18]. The primary antibodies used in the study, including rabbit anti-HGF (ab83760, Abcam, Cambridge, MA, USA), rabbit anti-p-MET (Tyr1234/1235) (3129; Cell Signaling Technology, Beverly, MA, USA), mouse anti-MET (sc-8057; Santa Cruz Biotechnology, Santa Cruz, CA, USA), mouse anti-VEGF (sc-7269, Santa Cruz Biotechnology), rabbit anti-p-VEGFR2 (Y951) (4991; Cell Signaling Technology), mouse anti-VEGFR2 (sc-393163; Santa Cruz Biotechnology), and mouse anti-glyceraldehyde-3-phosphate dehydrogenase (GAPDH) (sc-365062; Santa Cruz Biotechnology), were incubated with the blots overnight at 4°C. After incubation with the IRDye 680RD and IRDye 800CW secondary IgG (1 : 10,000; Sigma-Aldrich) for 2 h at room temperature, the blots were developed and quantified with the Odyssey Infrared Imaging System (Li-Cor Biosciences, Lincoln, NE, USA).

### 2.5. Immunohistochemistry

At day 7 following CNV (*n* = 5), the mice were killed, and the eyes were processed for cryosectioning. The cryosections (5 *μ*m) were stained with Collagen IV and p-MET or Collagen IV and p-VEGFR2. The primary antibodies, rabbit anti-Collagen IV antibody (ab6586; Abcam, Cambridge, MA, USA), rabbit anti-p-MET, anti-glial fibrillary acidic protein (GFAP) (sc-33673; Santa Cruz Biotechnology), and rabbit anti-p-VEGFR2 antibodies, were incubated at 1 : 100 in PBS at 4°C overnight. After rinsing with PBS, the specimens were treated with the secondary antibodies rhodamine-conjugated anti-rabbit IgG (1 : 100 in PBS; Life Technologies Corporation, Carlsbad, CA, USA) for Collagen IV and GFAP staining and Alexa Fluor 488-conjugated anti-mouse IgG (1 : 200 in PBS; R37120, Thermo Fisher, Carlsbad, CA, USA) for p-MET or p-VEGFR2 staining. The sections were stained with DAPI (1 : 1000; D1306, Molecular Probes, Eugene, OR, USA) and were observed under a confocal microscope.

### 2.6. Fundus Angiography

To determine CNV leakage and area, fundus fluorescein angiography (FFA) and indocyanine green angiography (ICGA) in mice were performed 7 d or 14 d after CNV. The mice were anesthetized, their pupils were dilated, and then the mice were intraperitoneally injected with fluorescein AK-FLUOR (17478025310, Akorn, Lake Forest, IL, USA) at 5 *μ*g/g body weight and indocyanine green (ICG) (1340009, Merck, St. Louis, MO, USA) at 0.075 *μ*g/g body weight. Fluorescent fundus images were acquired using a retinal imaging microscope (Micron IV, Phoenix Research Laboratories) 5 min and 10 min after fluorescein injection. Additionally, fluorescein leakage was graded by two independent masked observers using previously established criteria [19]. The total CNV area was measured in ICGA images via ImageJ software.

### 2.7. Immunohistochemistry in Choroidal Flat-Mounts

On day 7 or day 14 after CNV, the cornea and crystalline lens were removed from the enucleated eye, followed by radial relaxing incision in the eye cup of the choroid and sclera to perform immunostaining in choroidal flat-mounts [20]. The choroidal tissue specimens were stained with isolectin-B4 (IB4) or IB4 and phalloidin. The specimens were then incubated with a 1-mg/ml solution of Alexa Fluor 488-conjugated IB4 (I21411) and Alexa Fluor 568-conjugated phalloidin (A12380) antibodies prepared in ICC buffer at 4°C for 24 h. Both antibodies were from Thermo Fisher.

### 2.8. Hematoxylin and Eosin (HE) Staining of Paraffin Sections

After each animal was sacrificed by cervical dislocation, the eyeballs were enucleated and fixed with 4% PFA and then were conventionally dehydrated and embedded into paraffin wax for HE staining [21]. The analysis of the ratio of A (the distance from the ganglion cell layer to the outer edge of the inner nuclear layer) to B (the distance from the ganglion cell layer to the outer edge of the outer nuclear layer) was performed using Image J software (NIH, Bethesda, MD, USA).

### 2.9. Terminal Deoxynucleotidyl Transferase-Mediated dUTP Nick End-Labeling (TUNEL) Assay

TUNEL assay was performed on mouse eye 5 *μ*m cryosections [22].

### 2.10. Cell Culture and Treatment

The murine brain microvascular endothelial cell line b-End3 was obtained from American Type Culture Collection (ATCC; CRL-2299; Manassas, VA, USA). The cells were cultured in Dulbecco's modified Eagle's medium (DMEM) (Sigma-Aldrich, St. Louis, MO, USA) containing 1 g/l of glucose supplemented with 10% fetal bovine serum (FBS), 1% nonessential amino acids, 100 IU/ml of penicillin, and 100 *μ*g/ml of streptomycin (Sigma-Aldrich) at 37°C with 5% CO_2_. B-End3 cells were treated with dimethyloxalylglycine (DMOG; Sigma) diluted in 0.5% volume/volume (v/v) of dimethyl sulfoxide (DMSO, diluted in PBS) at 0.4 mM (hypoxia group) or the same volume of 0.5% DMSO (DMSO group) for 6 h. The b-End3 cells in the CBZ group were treated with 10 mM CBZ for 6 h (the same time as that for DMOG treatment).

### 2.11. 5-Ethynyl-2-deoxyuridine (EdU) Assay

The EdU kit (Cell Light EdU DNA imaging Kit, RiboBio, Guangzhou, China) was used to measure b-End3 cell proliferation [23]. The images were detected and analyzed using a confocal microscope (Olympus IX70, Tokyo, Japan). The average ratio of EdU-stained cells (red fluorescence) versus DAPI-stained cells (blue fluorescence) was used to assess b-End3 cell proliferation.

### 2.12. Transwell Migration Assay

The b-End3 cells (2.5 × 10^5^) were suspended in 250 *μ*l of serum-free DMEM and were seeded in the top chambers of 24-well Transwell plates (Corning Inc., Corning, NY, USA) to perform the Transwell migration assay [24]. ImageJ software was used to obtain an average cell count of the four stained membrane images.

### 2.13. Tube Formation Assay

Briefly, 50 *μ*l of growth factor-reduced Matrigel (BD Biosciences, Bedford, MA, USA) was coated into a 96-well plate and incubated at 37°C for 30 min. B-End3 cells (2 × 10^4^) were seeded on the Matrigel and treated for 6 h. Images were acquired at 5 randomly chosen microscopic fields. The number of closed tubes was analyzed using Olympus IX70 under ×200 magnification.

### 2.14. Statistical Analysis

The ISV length and absence ratio of ISV were measured using Imaris (version 7.2.3). These data were statistically analyzed using GraphPad Prism 5. All the data were displayed as means ± SEM. Statistical analysis was performed using one-way analysis of variance (ANOVA) (*P* < 0.05).

## 3. Results

### 3.1. CBZ Blocks Zebrafish Embryonic Angiogenesis without Obvious Neurodevelopment Impairment

**Figure 1:**
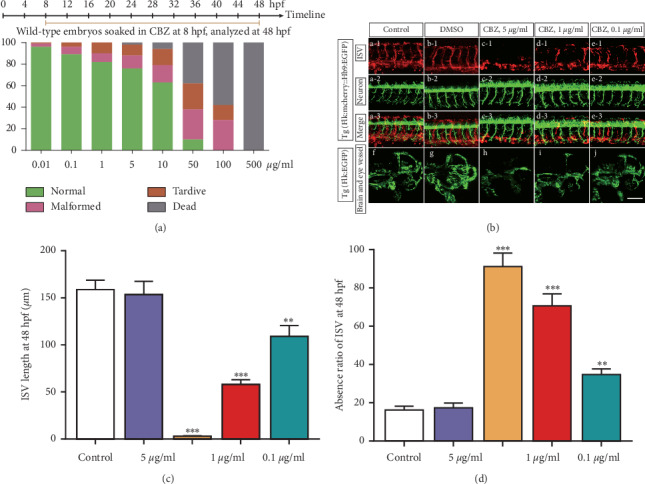
CBZ blocks zebrafish embryonic angiogenesis without obvious neurodevelopment impairment. (a) Percentage of the phenotype induced by 0.01, 0.1, 1, 5, 10, 50, 100, or 500 *μ*g/ml CBZ treatment at 8 hpf and analyzed at 48 hpf. The percentages of normal, malformed, tardive, and dead embryos are displayed in the green, pink, orange, and gray columns, respectively. (b) (a-1, b-1, c-1, and d-1) mCherry positive (red) ISV phenotypes are shown in the control, 0.1% DMSO, 0.1-*μ*g/ml CBZ, 1-*μ*g/ml CBZ, and 5-*μ*g/ml CBZ groups. Scale bar = 100 *μ*m. (b) (a-2, b-2, c-2, and d-2) EGFP-positive (green) cells in the neural tubes of the control group and 0.1, 1, and 5 *μ*g/ml CBZ groups. Scale bar = 100 *μ*m. (b) (a-3, b-3, c-3, and d-3) The merged images of ISV and neural tubes are shown. Scale bar = 100 *μ*m. (c, d) Statistical analyses of the ISV length and ISV absence ratio of each ISV in the control group and 0.1, 1, and 5 *μ*g/ml CBZ groups. *n* = 8/group. (b) (e, f, g, and h) EGFP-positive (green) brain and eye vessels of normal and 5, 1, and 0.1 *μ*g/ml CBZ groups. Scale bar = 100 *μ*m in Figures 1(c)–1(e). *n* = 8/group. ^*∗∗*^*P* < 0.001, ^*∗∗∗*^*P* < 0.005 vs. the control group.

Over the past decade, zebrafish has emerged as an established model to study multiple human ophthalmological disorders, including AMD, and screen antiangiogenic drugs [25, 26]. To identify the suitable dose of CBZ, we treated wild-type (WT) zebrafish embryos with 0.01, 0.1, 1, 5, 10, 50, 100, or 500 *μ*g/ml CBZ solution from the gastrula period (8 hpf) to pharyngula period (48 hpf) in triplicate (Figure 1(a)). CBZ treatment at concentrations beyond 5 *μ*g/ml caused some embryo death (Figure 1(a)). CBZ treatment at a concentration of 500 *μ*g/ml resulted in all dead embryos (Figure 1(a)). Some of the embryos with CBZ treatment at concentrations less than 50 *μ*g/ml were normal (Figure 1(a)). Because CBZ treatment at concentrations higher than 5 *μ*g/ml caused very severe phenotypes, including malformed, tardive, and dead, we selected 0.1, 1, and 5 *μ*g/ml as the working concentrations in subsequent experiments.

**Figure 2:**
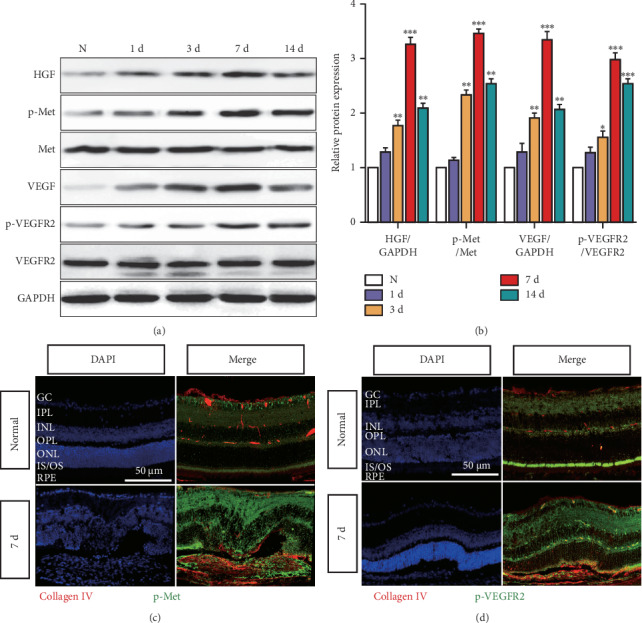
The phosphorylation of CBZ target molecules MET and VEGFR2 increases in the mouse CNV region. The mouse CNV model was generated by laser photocoagulation. (a) The HGF, p-MET, MET, VEGF, p-VEGFR2, and VEGFR2 protein levels were detected by western blotting in the normal group or CNV at 1, 3, 7, and 14 d groups. (b) Quantification of the HGF, p-MET, VEGF, and p-VEGFR2 protein levels in each group. n = 6/group, ^*∗*^*P* < 0.05, ^*∗∗*^*P* < 0.01, and ^*∗∗∗*^*P* < 0.005 vs. the normal group. Immunofluorescent staining of DAPI (nucleus), Collagen IV, and p-MET (c) or p-VEGFR2 (d) was performed in the normal and CNV 7 d groups. Scale bar = 50 *μ*m. n = 8/group (RPE, retinal pigment epithelium; OS, outer segment; IS: inner segment; ONL, outer nuclear layer; OPL, outer plexiform layer; INL, inner nuclear layer; IPL, inner plexiform layer; GC, ganglion cell layer).

To verify the effects of CBZ on embryonic angiogenesis and neurodevelopment in zebrafish, we used the transgenic line Tg (Flk:mcherry:Hb9:EGFP), in which vascular endothelial cells (VECs) and different motor neurons were marked with mCherry and EGFP, respectively. CBZ treatment at 0.1, 1, and 5 *μ*g/ml significantly inhibited ISV branching angiogenesis (Figure 1(b), a-1, b-1, c-1, and d-1). In particular, in the transgenic line Tg (Flk:EGFP), the zebrafish embryonic brain and eye vessels were impaired in the CBZ treatment groups (Figures 1(b), e–1(h)). The ISV length was decreased (Figure 1(c)) and the ISV absence ratio was increased in the CBZ treatment groups in a dose-dependent manner (Figure 1(d)). Moreover, CBZ treatment showed no neurodevelopment impairment of zebrafish embryos (Figure 1(b), a-2, b-2, c-2, and d-2). The data suggest that CBZ inhibits the angiogenesis of zebrafish embryos without affecting their neurodevelopment.

### 3.2. Phosphorylation of CBZ Target Molecules MET and VEGFR2 Increases in the Mouse CNV Region

CBZ acts as a receptor TKI with activities over a broad range of targets, including MET and VEGFR2 [6]. Therefore, we used laser photocoagulation to construct a mouse CNV model and western blotting to detect the HGF, p-MET, VEGF, and p-VEGFR2 protein levels in the RPE-choroid mix. The levels of these proteins were increased from 3 d following laser injury, peaking at 7 d. Thereafter, the expression levels of HGF, p-MET, and VEGF were decreased while p-VEGFR2 maintained a high level until 14 d (Figure 2(a) and 2(b)). Immunofluorescent staining also revealed that p-MET and p-VEGFR2 levels were increased at 7 d following laser injury and they colocalized with Collagen IV (a vascular basement membrane marker) (Figures 2(c) and 2(d)). The results suggest that the phosphorylation of CBZ target molecules, including MET and VEGFR2, is increased in the mouse CNV region.

### 3.3. CBZ Intravitreal Injection Alleviates CNV Leakage and the CNV Lesion Area and Downregulates the Phosphorylation of MET and VEGFR2

**Figure 3:**
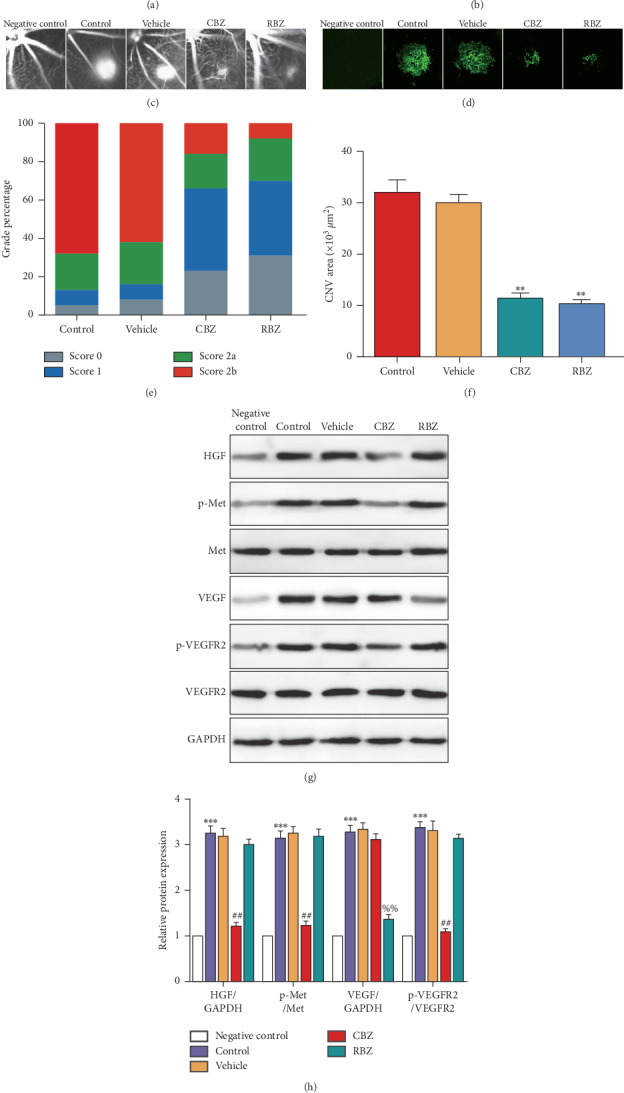
CBZ intravitreal injection alleviates CNV leakage and the CNV lesion area and downregulates the phosphorylation of MET and VEGFR2. (a) Schematic diagram of the experimental procedure. The mice were left untreated (with CBZ treatment, negative control) or were treated (control group) with a laser at day 0. At day 3, 0.1% DMSO (vehicle group), 2 *μ*g/*μ*l CBZ (CBZ group), or 10 *μ*g/*μ*l RBZ (RBZ group) (1 *μ*l for each) was injected intravitreally. The mouse eye tissues were collected and analyzed at day 7. (b) FFA was performed, and (e) fluorescein leakage in CNV lesions was graded at 7 d after CNV in negative control (*n* = 30 lesions), control (*n* = 32 lesions), vehicle (*n* = 30 lesions), CBZ (*n* = 32 lesions), and RBZ (*n* = 32 lesions) groups. Scale bar = 200 *μ*m. (c) ICGA was performed in the negative control (*n* = 30 lesions), control (*n* = 32 lesions), vehicle (*n* = 30 lesions), CBZ (*n* = 32 lesions), and RBZ (*n* = 32 lesions) groups. Scale bar = 200 *μ*m. (d, f) The mouse CNV lesion area at 7 d after CNV induction was assessed by the staining of choroidal flat-mounts with fluorescent IB4 in the negative control, control, vehicle, CBZ, and RBZ groups. Scale bar = 100 *μ*m. *n* = 8/group. ^*∗∗*^*P* < 0.01 vs. the control group. (g) The HGF, p-MET, MET, VEGF, p-VEGFR2, and VEGFR2 protein levels in the negative control, control, vehicle, CBZ, and RBZ groups were detected by western blotting. (h) Quantification of the HGF, p-MET, VEGF, and p-VEGFR2 protein levels in each group. *n* = 6/group. ^*∗∗∗*^*P* < 0.005 vs. the negative control group; ^##^*P* < 0.01 and ^%%^*P* < 0.01 vs. the control group.

To identify the role and mechanism of CBZ in treating CNV, CBZ intravitreal injection was performed 3 d following laser injury and analysis was carried out at 7 d (Figure 3(a)). ICGA and FFA showed decreased CNV leakage in the CBZ and RBZ groups (Figures 3(b) and 3(c)). Leakage score analysis also showed that the grade percentage scores of 0 and score 1 were increased, while that of score 2b was decreased in the CBZ and RBZ groups (Figure 3(c)). IB4 immunofluorescent staining indicated that the CNV lesion area was decreased in the CBZ and RBZ groups (Figures 3(d) and 3(e)). Additionally, CBZ downregulated the p-MET and p-VEGFR2 protein levels in the CNV group at 7 d, but RBZ did not affect p-MET and p-VEGFR2 expression (Figures 3(f) and 3(g)). The data showed that CBZ used in the mice without laser coagulation did not affect vascular leakage, vascular formation, and the expression of proangiogenic molecules (the negative control group in Figure 3). The results suggest that CBZ intravitreal injection alleviates CNV leakage and the CNV lesion area, likely by inhibiting the phosphorylation of MET and VEGFR2.

### 3.4. CBZ Shows No Obvious Intraocular Toxicity

**Figure 4:**
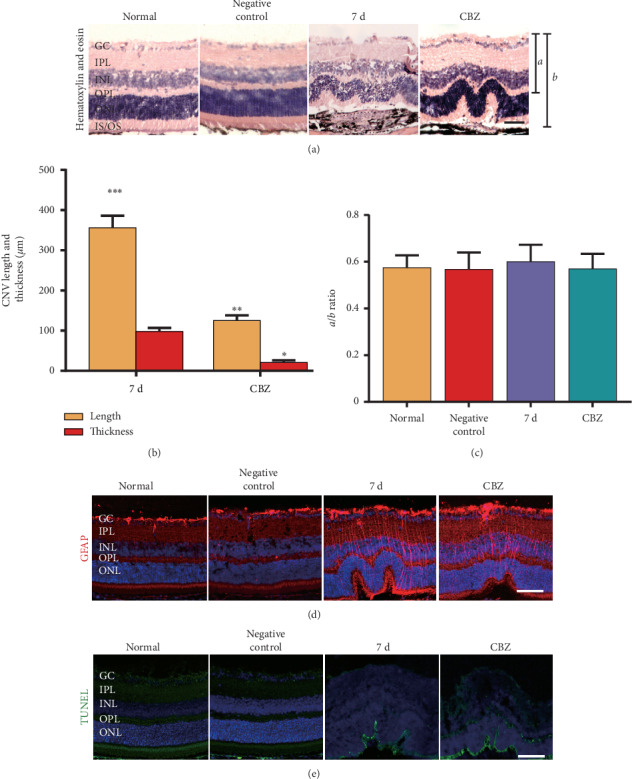
CBZ shows no obvious intraocular toxicity. (a) Mouse choroid-RPE-retina complex was performed HE stain in normal, negative control, CNV 7 d and CNV 7 d plus CBZ groups. (b) Quantification analysis of CNV length and thickness. ^*∗*^*P* < 0.05, ^*∗∗*^*P* < 0.01 vs. CNV 7 d group. (c) Quantification of the ratio of *a* to *b* displayed in Figure 4(a) was shown. (d) Immunofluorescent staining of DAPI (nucleus, blue), and GFAP (red) performed in the normal, negative control, CNV 7 d, and CNV 7 d plus CBZ groups. (e) Immunofluorescent staining of TUNEL (green), and DAPI (blue) on mouse choroid-RPE-retina cryosections in normal, negative control, CNV 7 d, and CNV 7 d plus CBZ groups. Scale bar = 50 *μ*m; *n* = 8/each group.

The examination of HE-stained retinal sections (Figure 4(a)) and quantification of the retinal thickness (Figures 4(b), and 4(c)) revealed no change in histologic morphology or retinal thickness between normal and CBZ-treated eyes. Moreover, CBZ treatment of the mice without laser coagulation showed no effect on the structure of the retina (Figures 4(a) and 4(c)). These data suggest that CBZ alleviates mouse CNV formation in the absence of intraocular toxicity. Meanwhile, GFAP (a Müller cell marker) [27] expression showed no increase in the CNV plus CBZ group at 7 d compared with that in the CNV group at 7 d (Figure 4(d)), indicating that CBZ has no toxic effect on Müller cells. Similarly, CBZ treatment did not increase cellular apoptosis (Figure 4(e)), showing that CBZ does not facilitate cellular apoptosis. Meanwhile, normal mice exposed to CBZ showed no difference in Müller cell damage and cellular apoptosis compared with the normal group (Figures 4(d) and 4(e)). The above data suggest that CBZ shows no intraocular toxicity, including histologic morphology change, Müller cell activation, and cellular apoptosis induction.

### 3.5. CBZ Oral Gavage Mitigates CNV Leakage and the CNV Lesion Area and Restrains the Phosphorylation of MET and VEGFR2

**Figure 5:**
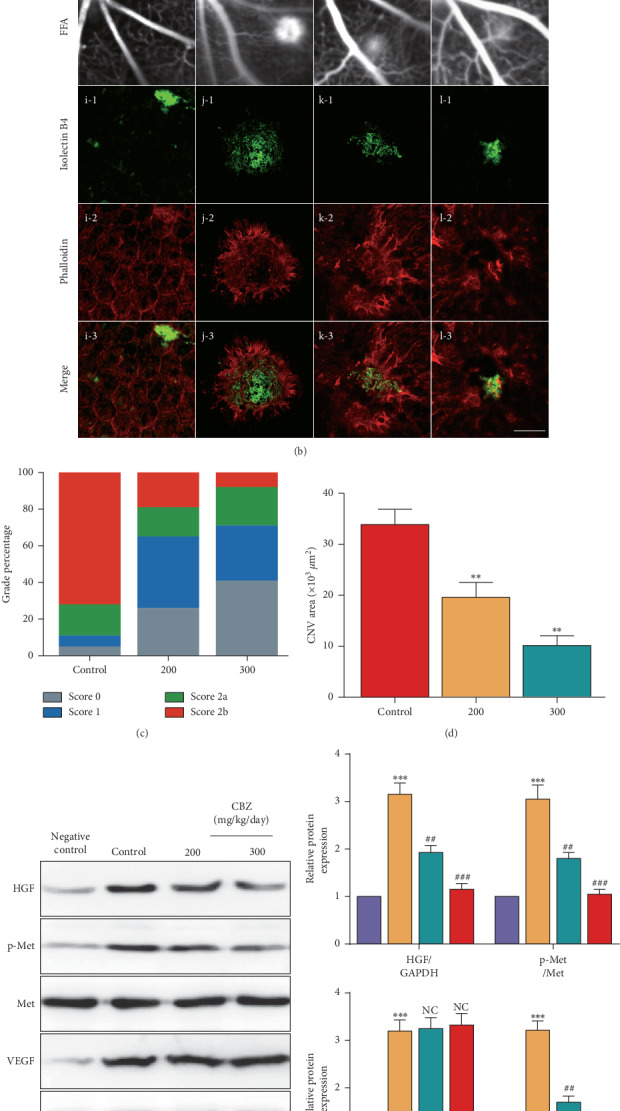
CBZ oral gavage mitigates CNV leakage and the CNV lesion area and restrains the phosphorylation of MET and VEGFR2. (a) Schematic diagram of the experimental procedure. The mice were left untreated (300 mg/kg/day CBZ, negative control) or were treated (control group) with a laser at day 0. Additionally, 200 or 300 mg/kg/day of CBZ was added to mouse daily fodder until day 14 when the mouse eye tissues were collected and analyzed. ICGA (b) (a, b, c, and c) and FFA (b) (e, f, g, and h) were performed, and (c) fluorescein leakage in CNV lesions was graded at 14 d after CNV in the control (*n* = 32 lesions), 200 mg/kg/day CBZ (*n* = 30 lesions), and 300 mg/kg/day CBZ (*n* = 32 lesions) groups. (b, d) (i-1, j-1, k-1, l-1, i-2, j-2, k-2, l-2, i-3, j-3, k-3, l-3) The mouse CNV lesion area 7 d after CNV induction was assessed by the staining of choroidal flat-mounts with fluorescent IB4 and phalloidin. Scale bar = 100 *μ*m. *n* = 8/group. ^*∗∗*^*P* < 0.01 vs. the control group. (e) The protein levels of HGF, p-MET, MET, VEGF, p-VEGFR2, and p-VEGFR2 in the negative control, control, 200 mg/kg/day CBZ, and 300 mg/kg/day CBZ groups were detected by Western blotting. (f) Quantification of the HGF, p-MET, VEGF, and p-VEGFR2 protein levels in each group. *n* = 6/group. ^*∗∗∗*^*P* < 0.005 vs. the negative control group; ^##^*P* < 0.01 and ^###^*P* < 0.005 vs. the control group; NS, not significant.

To further confirm the role of CBZ in the alleviation of CNV, CBZ oral gavage at the dose of 200 or 300 mg/kg/day was performed on the same day of laser injury and analysis was performed at 14 d (Figure 5(a)). FFA showed decreased CNV leakage in the CBZ groups (Figure 5(b), a, b, and c). Leakage score analysis also showed that the grade percentage of score 0 and score 1 increased, while the grade percentage of score 2b was decreased in the CBZ groups (Figure 5(c)). IB4 and phalloidin double staining indicated that the CNV lesion area was decreased in the CBZ groups (Figure 5(b) (d-3, e-3, f-3) and 5(d)). Additionally, CBZ oral gavage downregulated the HGF, p-MET, and p-VEGFR2 protein levels in CNV in the 14 d groups (Figures 5(e) and 5(f)). Furthermore, CBZ showed no effect on vascular leakage and formation in the normal mice (Figure 5(b)). The results suggest that CBZ oral gavage mitigates CNV leakage and the CNV lesion area via restraining the phosphorylation of MET and VEGFR2.

### 3.6. CBZ Decreases p-MET and p-VEGFR2 Expression, as well as Inhibits the Proliferation, Migration, and Tube Formation of b-End3 Cells In Vitro

**Figure 6:**
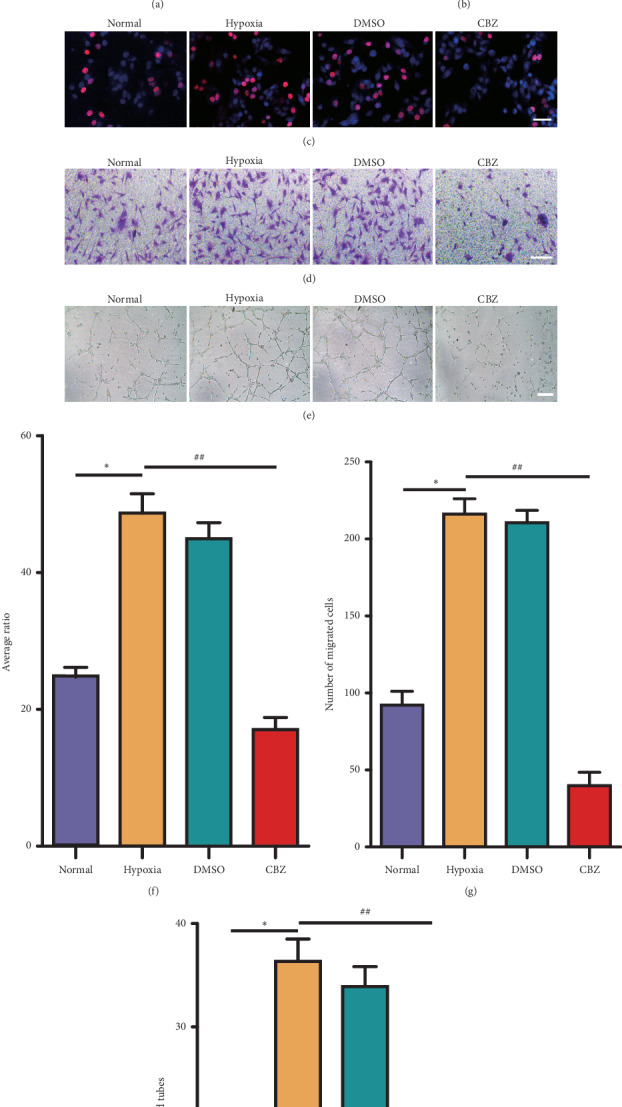
CBZ decreases p-MET and p-VEGFR2 expression, as well as inhibits the proliferation, migration, and tube formation of b-End3 cells *in vitro*. B-End3 cells were randomly divided into four groups—normal, hypoxia (0.4 mM DMOG treatment for 6 h), hypoxia plus DMSO (0.5% DMSO treatment for 6 h), and hypoxia plus CBZ (10 *μ*M CBZ treatment for 6 h) groups. (a) The HGF, p-MET, MET, VEGF, p-VEGFR2, and VEGFR2 protein levels were detected by Western blotting. (b) Quantification of the HGF, p-MET, VEGF, and p-VEGFR2 protein levels in each group. ^*∗∗∗*^*P* < 0.001 vs. the normal group. ^###^*P* < 0.001 vs. the hypoxia group. NS, not significant. (c) The EdU assay was performed to measure the proliferation capability of b-End3 cells. (f) The EdU-positive cell ratio was analyzed. ^*∗*^*P* < 0.05 vs. the normal group. ^##^*P* < 0.01 vs. the hypoxia group. (d) The Transwell assay was performed to detect the migration capability of b-End3 cells. (g) The number of migrated cells was analyzed. ^*∗*^*P* < 0.05 vs. the normal group. ^##^*P* < 0.01 vs. the hypoxia group. (e) The tube formation assay was performed. (h) The number of closed tubes was analyzed. ^*∗*^*P* < 0.05 vs. the normal group. ^##^*P* < 0.01 vs. the hypoxia group. (c–e) Scale bar = 100 *μ*m. *n* = 5/group.

To further identify the antiangiogenesis role of CBZ in choroid microvascular endothelial cells, mouse brain microvascular endothelial cell line b-End3 cells were exposed to hypoxia and CBZ to mimic CBZ therapy for mouse CNV [28]. CBZ downregulated HGF, p-MET, and p-VEGFR2 protein levels under hypoxia (Figure 6(a) and 6(b)). Furthermore, CBZ decreased the EdU-positive cell ratio (Figures 6(c) and 6(f)), number of migrated cells (Figures 6(d) and 6(g)), and number of closed tubes compared with those in the hypoxia group (Figures 6(e) and 6(h)), showing that CBZ inhibited the proliferation, migration, and tube formation of b-End3 cells *in vitro*. The data suggest that CBZ decreases p-MET and p-VEGFR2 expression, as well as inhibiting the proliferation, migration, and tube formation of b-End3 cells *in vitro*.

## 4. Discussion

CBZ promotes the apoptosis of hepatocellular carcinoma (HCC) [29], pancreatic ductal adenocarcinoma (PDA) [30], and melanoma cells [6]. However, we found that CBZ at 0.1, 1, and 5 *μ*g/ml hinders ISV formation without neurodevelopment impairment in zebrafish embryos, an effect that is different from that of combretastatin A-4 (CA-4) at 20 ng/ml that induces significant cell apoptosis in the central nervous system (CNS) of zebrafish embryos and adults [31]. However, a previous study revealed that 38% of patients with medullary thyroid cancer (MTC) treated with oral CBZ 175–265 mg/day for at least 4 months develop grade 1/2 peripheral neuropathy [32]. Therefore, the side effects of CBZ on the peripheral and central nervous systems require future studies.

In our study, we found that CBZ intravitreal injection or oral gavage mitigated CNV leakage and the CNV lesion area might via restraining the phosphorylation of MET and VEGFR2. Besides these mechanisms, autophagy functions to induce the degradation and elimination of unwanted intracellular components. A link exists between autophagy and proteasome-mediated proteolysis, which is upregulated upon exposure to various oxidative stimuli in AMD donor samples [33]. Impaired lysosomal function-triggered decreased autophagy flux may be a critical aspect of RPE cell degeneration and CNV development [34]. CBZ plays significant antitumor roles, including reduced tumor vascularity, increased autophagy, and altered cell metabolism, in a female athymic nude mouse colorectal cancer patient-derived tumor xenograft model [35]. Thus, we speculate that CBZ inhibits CNV formation via upregulating autophagy flux in the choroid.

To date, receptor tyrosine kinase (RTK) inhibitor family molecules have been used for the therapy of multiple cancers, including lung cancer [36], renal cancer [37], and cholangiocarcinoma [38]. Interestingly, our previous study found that the receptor tyrosine kinase (RTK) inhibitor brivanib downregulates the phosphorylation of FGFR1 and VEGFR2 and alleviates the leakage, lesion area, and formation of CNV via intravitreal injection or oral gavage [16]. *In vitro*, brivanib inhibits the hypoxia-induced proliferation, migration, and tube formation of microvascular endothelial cells. CBZ is also an RTK inhibitor that inhibits the phosphorylation of MET and VEGFR2, thereby mitigating the formation of CNV lesions. The inhibitory effect on the phosphorylation of VEGFR2 is a common mechanism for brivanib and CBZ in CNV, and other common mechanisms for these two RTK inhibitors need further investigation.

MET is an RTK that can be dysregulated by gene mutation, gene amplification, and protein overexpression through a ligand-dependent autocrine or paracrine loop [39]. Increased total MET and p-MET are associated with a worse outcome in renal cell carcinoma (RCC) via promoting angiogenesis [40]. The activation of MET can drive lymphangiogenesis, leading to lymph node metastasis of a mouse mammary tumor model [41]. Furthermore, the MET inhibitor KRC-408 suppresses the cell proliferation and angiogenesis of gastric cancer [42]. In the study, we also found that the p-MET protein level is increased in the mouse laser-induced CNV model, along with the localization of p-MET in the CNV region. CBZ intravitreal injection or oral gavage downregulated the p-MET protein level. These data suggest that MET might promote angiogenesis in the pathogenesis of CNV.

CBZ is a receptor TKI with activities against several targets, including MET, VEGFR2, Ret receptor tyrosine kinase (RET), Axl receptor tyrosine kinase (AXL), FMS-like tyrosine kinase 3 (FLT3), and kit receptor tyrosine kinase (KIT) [43]. The activities of CBZ towards multiple tumor models have been detected in several preclinical studies. Importantly, CBZ decreases the metastasis potential and tumor invasiveness compared with placebo or other antitumor agents that target VEGFR and have no effect on MET. Therefore, CBZ is clinically approved for the treatment of medullary thyroid cancer (MTC) [44] and RCC in the second line [45]. FLT3, which is expressed on CD34-positive hematopoietic stem/progenitor cells, acts as a crucial receptor for both normal myeloid and lymphoid differentiation [46]. FLT3 phosphorylation activates the intracellular signaling pathways, contributing to cell proliferation [47]. Mounting evidence has indicated that inflammation, involving the infiltration of macrophages and microglia, plays an important role in the development of CNV [48, 49]. Mesenchymal stromal and circulating angiogenic cells repair the tissues by expanding CD34-positive cells [50]. Mice deficient in FLT3 or that receive an FLT3 inhibitor (AC220) show significantly reduced areas of laser-induced CNV [51]. Whether CBZ alleviates CNV progression via inhibiting the phosphorylation of FLT3 needs further exploration.

In summary, *in vivo* CBZ impedes zebrafish embryonic angiogenesis without neurodevelopment impairment, as well as alleviates mouse CNV leakage and the CNV lesion area, inhibits HGF expression, and inhibits MET and VEGFR2 phosphorylation. *In vitro*, CBZ alleviates the proliferation, migration, and tube formation of endothelial cells. However, whether CBZ relieves CNV via promoting autophagy will be resolved in a future study.

## Data Availability

The data used to support the findings of this study are available from the corresponding author upon request.
